# Supplementary material for: PchE Regulation of Escherichia coli O157:H7 Flagella, Controlling the Transition to Host Cell Attachment
Source: Int J Mol Sci. 2020 Jun 28;21(13):4592. doi: 10.3390/ijms21134592 (PMC7369912; doi:10.3390/ijms21134592)
Supplement: Supplementary file 1 [file ijms-21-04592-s001.pdf]

Table S1. Primers used in this study.

Strain Name	Description
PA20	*E. coli* serotype O157:H7 clinical isolate
PA20Δ*pchE*	PA20 with deletion of *pchE*
PA20Δ*csgD*	PA20 with deletion of *csgD*
PA20Δ*csgBA*	PA20 with deletion of *csgB* and *csgA*
PA20Δ*fliC*	PA20 with deletion of *fliC*
PA20 SMX-TM	PA20 induced with 540 μg/L SMX and 108 μg/L TM
PA20 pSE380	PA20 transformed with plasmid pSE380
PA20 pSE380::*pchE*	PA20 transformed with plasmid pSE380 carrying cloned *pchE*
PA20Δ*fliC* pSE380	PA20Δ*fliC* transformed with plasmid pSE380
PA20Δ*fliC* pSE380::*fliC*	PA20Δ*fliC* transformed with plasmid pSE380 carrying cloned *fliC*
PA20Δ*pchE* pMLB1034::*pchE*	PA20Δ*pchE* transformed with plasmid pMLB1034 carrying cloned *pchE* promoter

Primer	Sequence	Purpose
pchE380F	aagccatggtgacggtattccgttctgag	PchE expression
pchE380F	aacgagctccggctgttgattagctgtctgg	PchE expression
RT-CsgDF	tggacgatatctcttcaggctc	qRT-PCR
RT-CsgDR	cgcggtacgggtaatcttcag	qRT-PCR
RT-CsgAF	gagctgaacatttaccagtacgg	qRT-PCR
RT-CsgAR	atcgattgagctgtcatctgagc	qRT-PCR
RT-H-NSF	aacggcggcaaggctattcag	qRT-PCR
RT-H-NSR	cgaacgtcgcgaagaagaaagc	qRT-PCR
RT-StpAF	gtgctaatttttcctggcgctc	qRT-PCR
RT-StpAR	atggctcgcgaattctccattg	qRT-PCR
RT-LerF	agtatatcccagctcttgtaagg	qRT-PCR
RT-LerR	gcaaattgcagttctacagcagg	qRT-PCR
RT-EaeF	tatcggcgttatccgctttagc	qRT-PCR
RT-EaeR	cctatgaccgtaatggcaatagc	qRT-PCR
RT-EspPF	gtaccatttcctgacttctcacc	qRT-PCR
RT-EspPR	gttttgcgtcgcacctgagtgg	qRT-PCR
RT-LpfA1F	ggatttgtcaccaaccgcagc	qRT-PCR
RT-LpfA1R	actgtattcgcagcagagtctg	qRT-PCR
RT-F9fimF	cgctaacgtatcgtcattactgg	qRT-PCR
RT-F9fimR	gttcttctcgcctggctaaagc	qRT-PCR
RT-FimDF	ctgtttgacgactgaaagcctg	qRT-PCR
RT-FimDR	cattaaaagttacgctggcatcc	qRT-PCR
RT-EcpAF	cgatcatcacggtatcgccag	qRT-PCR
RT- EcpAR	ggctatcgagggtgactcaac	qRT-PCR
RT-ppdDF	cgtcgcaggtatctaatccacc	qRT-PCR
RT-ppdDR	gcttatcaaaactacctgcgcaaag	qRT-PCR
RT-flhCF	tgctttgcagatgctggaaagc	qRT-PCR
RT-flhCR	tgagaatggcagcatgcctttc	qRT-PCR
RT-flhDF	cgacaacattagcggcactagc	qRT-PCR
RT-flhDR	ctggtggctgtcaaaacggaag	qRT-PCR
RT-FliAF	cctgctccagttgccctattg	qRT-PCR
RT-FliAR	aacttacgcagtgcagcgtatc	qRT-PCR
RT-FliCF	gtctttcgccagcacgttcac	qRT-PCR
RT-FliCR	gccactacagggactaactcc	qRT-PCR
RT-MotBF	ttgtcgtcaaacgacgcaaagc	qRT-PCR
RT-MotBR	agatggagatcagccacatcac	qRT-PCR
RT-CheAF	gaactgttggctgatatggagc	qRT-PCR
RT-CheAR	acgctgaagccaaaagttcctg	qRT-PCR
RT-CheRF	gaatctaatcagcacagcggtg	qRT-PCR
RT-CheRR	cgcgagcagagggaaatgatg	qRT-PCR
RT-gyraseF	gcctaaacgaataccgcgaac	qRT-PCR
RT-gyraseR	atgctgttctccgccgaagg	qRT-PCR

pchE_GSP1	gattacgccaagcttctgtgccttcctggaacattcc	RACE primer
pchE_GSP2	gattacgccaagcttaagcctttcgcctcgagttcttc	RACE primer
fliCred50F	gaaacccaatacgtaatcaacgacttgcaatataggataacgaatc atg	*fliC* deletion
fliCred50R	aggcaatttggcgttgccgtcagtctcagttaatcaggttacaacgat ta	*fliC* deletion
fliCproveF	cgacagacgataacaggtttgac	Confirm *fliC* deletion
fliCproveR	caattcaacttgtaggcctgataagc	Confirm *fliC* deletion
pchEred50F	aggcgatgacccttacggtgacggtattccgttctgagggggtggc gatgaattaaccctcactaaagggcg	*pchE* deletion
pchEred50R	ccataccgtttcaactggtgcaaaaaaagccggatttctccggctgtt gattataatacgactcactatagggctc	*pchE* deletion
15881034F	aagcccggggatgttccagcctgatgatgaag	PchE/LacZ fusion
15881034R	aagggatcccccatcgccacccccctcagaacgg	PchE/LacZ fusion
